# Social prediction modulates activity of macaque superior temporal cortex

**DOI:** 10.1126/sciadv.abh2392

**Published:** 2021-09-15

**Authors:** Lea Roumazeilles, Matthias Schurz, Mathilde Lojkiewiez, Lennart Verhagen, Urs Schüffelgen, Kevin Marche, Ali Mahmoodi, Andrew Emberton, Kelly Simpson, Olivier Joly, Mehdi Khamassi, Matthew F. S. Rushworth, Rogier B. Mars, Jérôme Sallet

**Affiliations:** 1 Wellcome Centre for Integrative Neuroimaging, Department of Experimental Psychology, University of Oxford, Oxford, UK.; 2 Institute of Psychology, University of Innsbruck, Innsbruck, Austria.; 3 Wellcome Centre for Integrative Neuroimaging Centre for Functional MRI of the Brain (FMRIB), Nuffield Department of Clinical Neurosciences, John Radcliffe Hospital, University of Oxford, Oxford, UK.; 4 Donders Institute for Brain, Cognition and Behaviour, Radboud University Nijmegen, Nijmegen, Netherlands.; 5 Biomedical Sciences Services, University of Oxford, Oxford, UK.; 6 Institute of Intelligent Systems and Robotics, Sorbonne Université, CNRS, Paris, France.; 7 Univ Lyon, Université Lyon 1, Inserm, Stem Cell and Brain Research Institute U1208, Bron, France.

## Abstract

The ability to attribute thoughts to others, also called theory of mind (TOM), has been extensively studied in humans; however, its evolutionary origins have been challenged. Computationally, the basis of TOM has been interpreted within the predictive coding framework and associated with activity in the temporoparietal junction (TPJ). Here, we revealed, using a nonlinguistic task and functional magnetic resonance imaging, that activity in a region of the macaque middle superior temporal cortex was specifically modulated by the predictability of social situations. As in human TPJ, this region could be distinguished from other temporal regions involved in face processing. Our result suggests the existence of a precursor for the TOM ability in the last common ancestor of human and Old World monkeys.

## Teaser

The macaque middle superior temporal sulcus shares functional properties with the human temporoparietal junction supporting mentalizing.

## INTRODUCTION

The ability to attribute mental representations to others, called theory of mind (TOM) (*1*), is key to complex human social interactions (*2*, *3*). While TOM’s neural bases have been extensively studied in humans, the question of its evolutionary origins has been disputed since the concept was first introduced (*1*, *2*, *4*, *5*).

Behavioral paradigms have been developed to specifically address the question of TOM ability in animals (*6*), but despite ingenious designs, the interpretation of performances on TOM-like tasks across primate species has been debated (*2*, *5*, *7*, *8*). Difficulties in addressing the question of TOM in animal models are partly due to the reliance of human TOM studies on linguistic stimuli (*9*). Two recent studies have nevertheless attempted to solve this problem, by designing innovative nonverbal false-belief task, a canonical test in the study of TOM (*10*). They showed that great apes and even Japanese macaques were able to anticipate other agents’ behaviors driven by false belief (*11*, *12*), suggesting the emergence of TOM abilities as ancient as the common ancestor of humans and Old World monkeys. Some authors, however, warn that such results should be considered cautiously (*13*).

Brain networks supporting TOM abilities in humans have been most notably identified as the medial prefrontal cortex (MPFC) and the temporoparietal junction (TPJ) (*9*, *14*). Both areas have been shown to undergo great expansion between the macaque and human brains (*15*), and the ensuing reorganization of the TPJ and posterior superior temporal sulcus (STS) between species is still unclear (*16*). However, MPFC has been shown to maintain a broadly similar anatomical organization in macaques and humans (*17*). Furthermore, using functional connectivity, a middle STS (midSTS) area was shown to share similar connectivity patterns with the human TPJ (*18*). Evidence of shared neuroanatomical properties suggests that the macaque MPFC and midSTS could share similar functions with the human MPFC and TPJ (*19*). In support of this hypothesis, both the MPFC and the midSTS have been associated with processing social information (*20*–*28*) and complex social interactions (*23*, *24*, *29*), although the question of TOM was not directly tested in these studies.

**Fig. 1.**
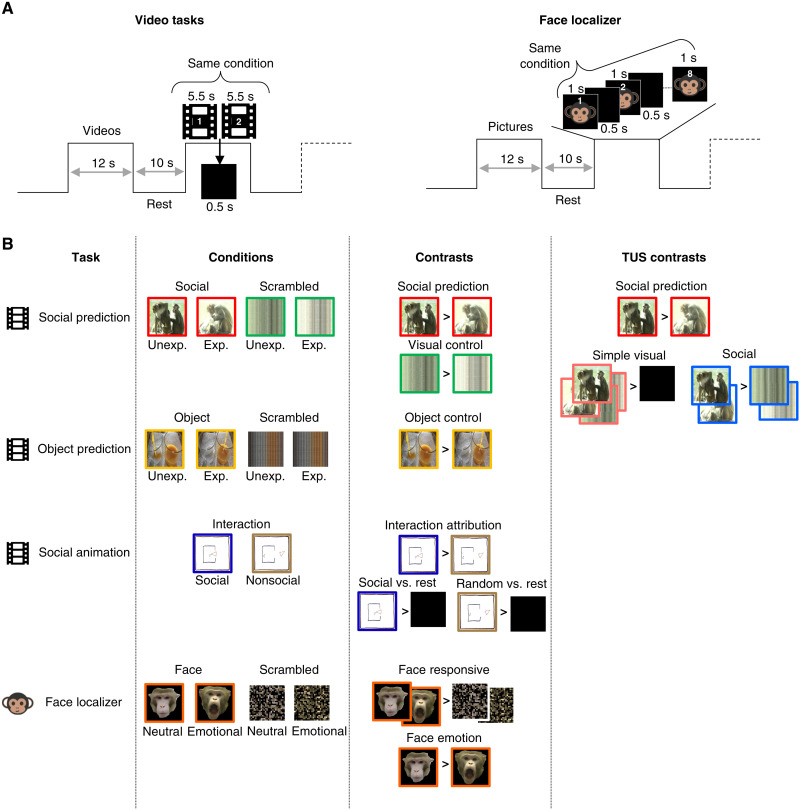
Summary of tasks used. (**A**) Schematic representing the timings for video-based tasks and the face localizer. Three blocks of each task are represented here. (**B**) Details of the tasks: Represented an example frame for each condition and contrasts of interest for the study as well as the control contrasts used after perturbation by transcranial ultrasound stimulation (TUS). Unexp., unexpected; Exp., expected. Photo credit for social prediction scenes and macaque faces: Jérôme Sallet, University of Oxford. Photo credit for object prediction scenes: Matthias Schurz, University of Oxford.

Theoretical developments in computational neuroscience propose an alternative method to compare human and animal social abilities. Rather than looking for TOM itself in other species, it may be profitable to seek evidence of more basic computational processes linked to TOM (*30*–*33*). Computational models describe human TPJ and MPFC activation during social tasks within a predictive coding framework (*3*, *34*). This framework predicts that deviations from expected social behaviors should lead to change of activity in these areas. It allowed us to design a nonlinguistic task for functional magnetic resonance imaging (fMRI) to investigate the existence of neural architecture supporting the computation of TOM in macaques and its relationship to other social circuits (Fig. 1).

## RESULTS

### Macaques’ midSTS is modulated by social expectation

**Fig. 2.**
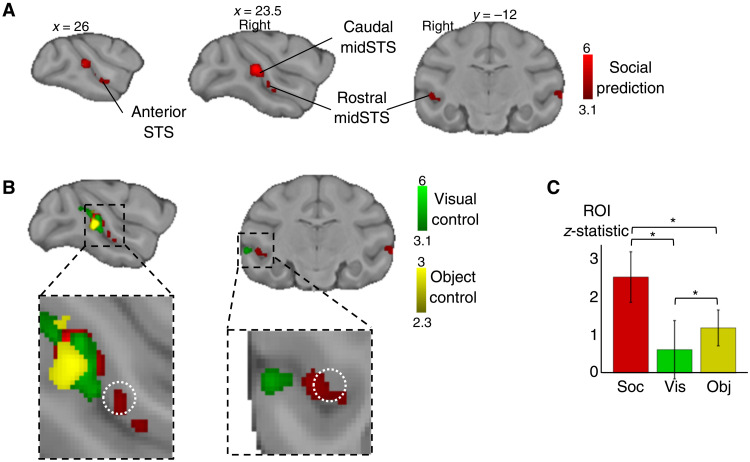
Modulation of macaque STS activity. (**A**) Social prediction: Group contrast of unexpected versus expected social situation revealed activity in rostral and caudal midSTS [*n* = 14, cluster-corrected at *z* > 3.1, *P* < 0.05 family-wise error (FWE)–corrected]. (**B**) Overlap between responses to social prediction and control conditions (visual control: *n* = 14, cluster-corrected at *z* > 3.1; object control: *n* = 7, cluster-corrected at *z* > 2.3 and both *P* < 0.05 FWE-corrected). The white dotted circle represents a macaque TPJ-like region identified previously (*18*). (**C**) Mean *z*-statistic obtained in the region of interest [ROI; white circle in (B)] for social prediction (Soc), visual control (Vis), and object control (Obj). Error bars represent SDs [* indicates significance (*P* < 0.05) in the Wilcoxon signed-rank test, Bonferroni corrected for multiple comparison; social × visual: *P* = 6 × 10^−43^; social × object: *P* = 3 × 10^−43^; object × visual: *P* < 1 × 10^−21^].

To investigate whether macaque brain areas signal deviation from social expectation, we presented 14 rhesus macaques with a free-viewing fMRI paradigm consisting of video clips of macaques interacting socially. This approach has been successfully used to identify brain networks supporting social cognition in macaques (*20*) but has not yet been used to identify computations supported in those circuits. In our videos, social situations either followed an expected scenario (e.g., continuous grooming or playing; movie S1) or were interrupted by an unexpected event (e.g., grooming or playing interrupted by a fight; movie S2). Several brain areas showed higher activation for the unexpected than expected social events, including regions belonging to the visual cortex and oculomotor-related regions (fig. S1 and table S1). Two clusters in the midSTS were also identified, which we will refer to as caudal midSTS and rostral midSTS (Fig. 2A and table S1). The rostral midSTS has often been associated with the macaque social brain (*18*, *21*, *35*).

To rule out explanations in terms of basic visual features, we first contrasted the neural response to scrambled videos of unexpected versus expected social situations, which were matched in terms of luminosity and movement to the original videos (visual control). The visual control contrast elicited higher activation in the caudal midSTS but not in the more rostral part of the midSTS (Fig. 2B, table S1, and fig. S2A). Unexpected social situation videos contain, by definition, more unexpected movement, and therefore, it is expected that this visual control would recruit regions in caudal midSTS that include the motion-sensitive areas MT (middle temporal area), FST (fundus of the superior temporal visual area), and MST (medial superior temporal area) (*35*, *36*).

We then tested the social specificity of the modulation of activity observed for social prediction in a subset of subjects (*n* = 7/14, object control) using nonsocial scenes containing inanimate objects. To match closely with the social situation videos, these videos were designed to represent situations with or without a departure from an expected and established physical regularity, such as the location, identity, or movement (movies S3 and S4). Regardless of whether we examined activity at the original threshold (*z* > 3.1) or at a more liberal threshold (*z* > 2.3) to account for the smaller number of animals, there was no evidence for activity in rostral midSTS but only in caudal midSTS for this object control (Fig. 2B, table S1, and fig. S2B). A conjunction analysis between the social prediction contrast and each of our two control conditions (fig. S2C) confirmed the specificity of the modulation of activity by social predictability in rostral midSTS cluster.

From here on, we will refer to this specific rostral midSTS region as “social prediction area” (SPA). It overlaps with cytoarchitectonically defined temporo-parieto-occipital association area and PG associated area of STS (*37*). From this location, we can also rule out an overlap with body-responsive areas that have been identified either posteriorly or ventrally to the SPA (*20*, *26*). It has also recently been shown that strategic social signaling in the rostral midSTS involves a different set of neurons than the ones responding to faces and bodies (*23*). The rostral midSTS that we identified corresponds to a midSTS region previously identified for its connectivity pattern most resembling that of human TPJ (*18*). In this independently defined region of interest (ROI), we observed that social prediction induced significantly higher activation than control conditions (Fig. 2C).

To confirm that the social prediction modulation in the SPA was not due to a thresholding effect and illustrate the specificity of its activity, we performed the three contrasts (social prediction, visual control, and object control) using the same independent ROI identified previously (*18*) to restrict the statistics. We observed a significant activation in the ROI only in the social prediction contrast and not the two others with a cluster correction (fig. S3, top). Because the extent of this ROI is quite small, we also performed voxel correction, which showed again the specificity of activation in this region for the social prediction contrast (181 voxels significant out of the 257 voxels of the ROI) and only a few voxels for the other two on the posterior edge of the ROI (12 for the visual control and 3 for the object control; fig. S3, bottom).

While we observed midSTS clusters bilaterally, some hemispheric differences were noticeable. The right caudal midSTS cluster, unlike the left caudal midSTS, extended toward the end of the STS, including V4t on its ventral bank (*37*). On the left hemisphere, the rostral midSTS cluster was located in a different area than the right SPA and had a more lateral position, extending from the dorsal bank of the STS to area TE on the lateral surface. To investigate whether the lack of social prediction modulation in the left SPA was indicative of a thresholding issue or a lateralized function, we defined a large ROI encompassing the whole STS around the coordinates of the previously mentioned midSTS region sharing neuroanatomical similarities with the human TPJ (*18*). With the same social prediction contrast but restricted on either the left or right hemisphere of this enlarged ROI, we found that a cluster survives the statistical correction in both hemispheres. Rather than a purely lateralized function, these results show that the modulation by social prediction in the SPA was bilateral but less robust in the left hemisphere (fig. S4).

### MidSTS modulation is robust to replication and disruption of oculomotor/attentional system

Last, we conducted a separate free-viewing experiment with a different set of four monkeys. Our first goal was to test for the robustness of the social prediction modulation in SPA in a replication study. Our second goal was to determine the impact of a disruption of the frontal eye field (FEF) on the computation performed by SPA. The FEF is usually associated with the attentional system, and connectivity at rest between the FEF and the STS has been reported (*17*, *38*). Because of the activity observed in the FEF in our social prediction contrast, we were concerned that a top-down attentional signal could mediate the modulation of activity observed in the SPA. Because of the passive nature of the task, it was not possible to causally and directly address the role of the midSTS. Instead, we used repetitive transcranial ultrasound stimulation (TUS) protocol to disturb brain activity over key ROIs, for at least 2 hours after stimulation (*39*), to check for potential confounding effects.

**Fig. 3.**
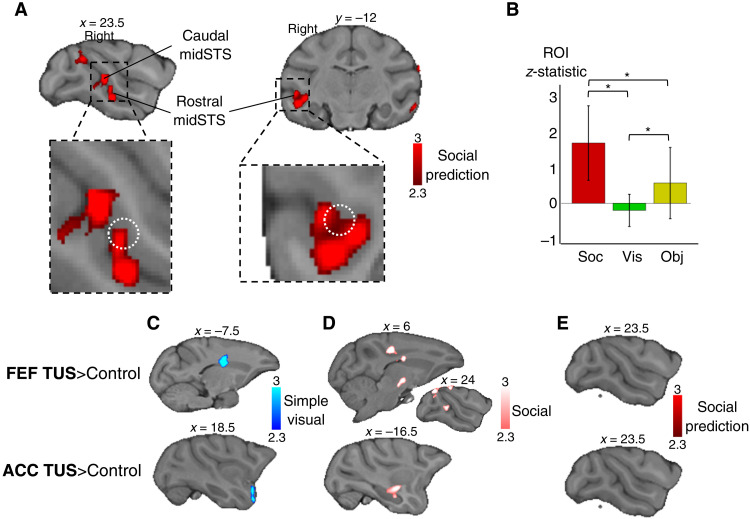
Replication of the modulation of macaque STS activity and effect of ultrasound stimulation. (**A**) Social prediction: Group contrast of unexpected versus expected social situation revealed activity in rostral and caudal midSTS (*n* = 4, cluster-corrected at *z* > 2.3, *P* < 0.05 FWE-corrected). Insets show the white dotted circle representing a macaque TPJ-like region identified previously (*18*). (**B**) Mean *z*-statistic obtained in the ROI (white circle) for social prediction (Soc), visual control (Vis), and object control (Obj). Error bars represent SDs [* indicates significance (*P* < 0.05) in the Wilcoxon signed-rank test, Bonferroni corrected for multiple comparison). (**C**) Simple visual: Two-sample paired *t* test for higher activation in FEF stimulation condition (top) or ACC (bottom) compared to control for the group contrast of videos versus black screen (*n* = 4, cluster-corrected at *z* > 2.3, *P* < 0.05 FWE-corrected). (**D**) For the group contrast of social videos versus scrambled videos (*n* = 4, cluster-corrected at *z* > 2.3, *P* < 0.05 FWE-corrected) (**E**) and for the social prediction contrast (*n* = 4, cluster-corrected at *z* > 2.3, *P* < 0.05 FWE-corrected, nothing significant).

We used the sessions without previous stimulation as a replication of the social prediction study, revealing the same rostral midSTS region as specifically modulated by social prediction (Fig. 3, A and B, and table S2). In the replication, the visual and object control contrasts did not yield any significant results in rostral midSTS and there was no conjunction with the social prediction contrast (fig. S5).

In both the original and replicated studies, we observed a cluster just anterior to the genu of the arcuate sulcus, an oculomotor region often referred to as the FEF. To rule out a putative attentional or oculomotor confound with the social prediction modulation, we used, before the awake fMRI data acquisition, a repetitive TUS of the FEF. In separate sessions, the anterior cingulate cortex (ACC), a region known for its role in social cognition (*20*–*22*), was targeted as an active control region. The efficacy of the stimulations was revealed by causal perturbation of activity in distant brain regions observed in two relevant contrasts: a simple visual contrast (videos versus black screen) and a social contrast (social videos versus scrambled) (fig. 3CD). However, in our contrast of interest—the social prediction—no difference between stimulation and nonstimulation sessions could be observed (Fig. 3E and fig. S5C). These results show that SPA was modulated by the predictability of social situation, independently of attentional or oculomotor effect led by the FEF. They confirmed the social specificity of the activity modulation in the SPA.

### Attribution of mental states to geometric shapes

We also presented animals with a nonlinguistic task used to study TOM in humans that is relying on animation of geometric shape acting either socially or randomly (*40*). Although this task has been criticized as not being a proper TOM task (*41*), discriminating between social and random interactions of the abstract shapes has been associated with modulation of activity in the vicinity of the TPJ and posterior STS in humans (*40*). In macaques, contrasting activity between the two types of videos did not reveal different brain activity in SPA. Contrasting each video type with rest blocks revealed that modulations of activity for both social and random interactions were located in visual areas, confirming that macaques were looking at the videos but did so similarly for both (fig. S6).

### Relationship of SPA with the face-responsive brain network

**Fig. 4.**
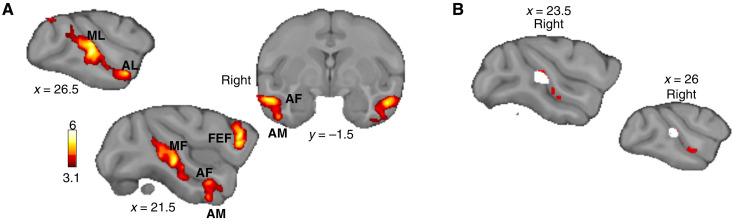
Face-responsive areas in macaques. (**A**) Macaque group contrast of face versus scrambled pictures (*n* = 14, cluster-corrected at *z* > 3.1, *P* < 0.05 FWE-corrected). (**B**) Conjunction analysis (white) of social prediction contrast activation (red) and face patches (cluster-corrected at *z* > 3.1, *P* < 0.05 FWE-corrected).

To further test the specificity of SPA responses and their relationship with known STS functions, we investigated how SPA is related to face patches, a set of face-responsive areas located in STS and inferotemporal cortex (*27*). We analyzed awake fMRI data from a face localizer collected in our initial group of 14 rhesus macaques. Our localizer consisted of pictures of neutral and emotional (e.g., lip-smacking and open mouth) macaque faces and their scrambled equivalent during fMRI. This method has been shown to identify the face-responsive brain regions as opposed to the face-selective brain regions by using a localizer combining face, body, and object pictures (*42*). In 12 of 14 animals, we were able to identify all six face patches previously reported (*27*, *43*): posterior lateral (PL), middle lateral (ML), middle fundus (MF), anterior lateral (AL), anterior fundus (AF), and anterior medial (AM) (Fig. 4A, fig. S7, and table S3).

A conjunction analysis revealed no significant overlap between face patches and SPA (Fig. 4B). At the single-subject level, we noticed that SPA peaks tended to be located in a more dorsal/fundus section of midSTS and, therefore, in a distinct cytoarchitectonic area compared to face patches (fig. S7). Our results are supported by recent findings showing that neurons in the ventral bank of the midSTS signal selectively cooperative social behavior, independently of visual sensitivity to faces and bodies (*23*).

**Fig. 5.**
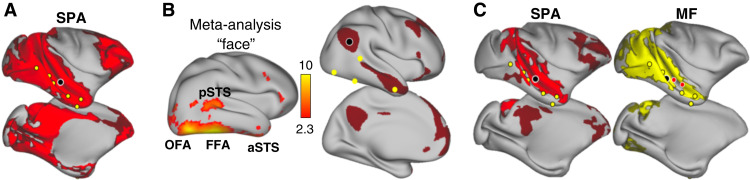
Face patch system and resting-state functional connectivity in macaques and humans. (**A**) Resting-state connectivity associated with SPA (black circle) from a full correlation to the whole brain (face patches are yellow circles). (**B**) Human comparison. Left: Meta-analysis results (Neurosynth) for “face,” displayed on the right hemisphere (pSTS, posterior STS; OFA, occipital face area; FFA, fusiform face area; aSTS, anterior STS). Right: Resting-state connectivity of TPJ (Cohen’s *d* effect size thresholded at 0.6). (**C**) Resting-state connectivity associated with SPA (black circle) from a partial correlation to the whole brain while accounting for face patches connectivity (left, red). Resting-state connectivity associated with MF (black circle) from a partial correlation to the whole brain while accounting for SPA connectivity (right, yellow; SPA and its anterior section are red circles). For all macaque resting state: *n* = 12, TFCE-corrected, FWE-corrected at *P* < 0.01 in bright color and 0.01 < *P* < 0.05 in dark color.

We then conducted a resting-state fMRI analysis to determine the relationship between the SPA and the face patches. We computed the functional connectivity profiles of macaques’ SPA with both full correlation as available in humans and a more specific partial correlation. The full correlation revealed that macaques’ SPA connectivity profile comprised face-responsive regions and other visual areas (Fig. 5A), but these were absent for human TPJ connectivity profile [coordinates from (*18*); Human Connectome Project (HCP) resting-state data (*44*); Fig. 5B]. However, computing the partial connectivity, by regressing out the time series of all face patches, reveals that SPA is specifically coupled with dorsal STS, posterior cingulate, and prefrontal cortex, resembling the human TPJ connectivity profile (Fig. 5C). Similarly, computing the partial connectivity of the face patches, by regressing out the time series of the SPA (and its anterior section), revealed a network involving mostly STS and the visual cortex (Fig. 5C). In summary, connectivity results not only provide further evidence for the distinction of face patch and SPA systems but also reveal stronger interactions between the two systems in macaques than in humans.

## DISCUSSION

Overall, our results revealed a brain region in macaques’ rostral midSTS that is specifically sensitive to expectation violation during free viewing of social scenes. Its location on the dorsal bank/fundus of the STS is compatible with a functional module identified as being responsive to natural social scenes (*45*) and strategic cooperation (*23*) but is distinct from previously identified modules associated with face, gaze following, or body patches (*26*–*28*, *43*). Here, we were able to characterize a computational property associated with this region. We interpret this response in a predictive coding framework providing the signature of the neurocomputational mechanism supporting mentalizing abilities in humans (*3*). Evidence for this type of coding has been uncovered in adjacent regions of the temporal cortex for processing nonsocial information in macaques (*46*). Furthermore, the midSTS region sensitive to prediction in the social domain corresponds to the region that was previously shown to share similar connectivity profiles with the human TPJ (*18*). Unlike in human studies (*9*, *14*), our social prediction analysis did not reveal any change of activity in macaque MPFC. This may reflect the nature of the passive free-viewing tasks compared to the active decision-making tasks used in humans (*34*, *47*).

Our results suggest an evolutionary trajectory in brain organization that in humans has resulted in area TPJ. The connectivity of face-responsive areas and the SPA differs in both humans and rhesus macaques, but the two circuits are more integrated in macaques; macaque SPA retains connectivity to face patches, while human TPJ shares little connectivity with the face-responsive system. On one hand, the stronger independence between TPJ and the visual system in humans might enable TOM computations to be applied to abstract information. On the other hand, midSTS interactions with the visual system in macaques might reflect stronger dependencies of TOM-related computations on visuo-social information. This constraint might explain why macaques did not distinguish between socially and randomly interacting abstract shape as previously observed (*48*) despite their abilities for face pareidolia (*49*). These between-species differences might reflect greater specialization of TPJ in humans that may have occurred in association with the expansion and reorganization (*16*) of the temporal cortex in the hominid evolution.

### Limitations and future studies

The debate around TOM and its evolutionary origins is a delicate one to tackle because of many hurdles faced in the design of tests and their interpretations. Our study, using a free-viewing paradigm, was designed in the computational framework of predictive coding rather than in the classical false-belief framework. Both approaches have been shown to recruit the mentalizing system in humans (*41*). As only one female was used here, future studies should seek to balance the ratio of male and female subjects. Although the same social prediction modulation in SPA was observed in males and in this female subject, possible sex differences cannot be ruled out. The videos for the free-viewing paradigm were selected to represent natural life events and therefore contain inherent variability in the context around the condition tested, such as the number of monkeys involved or the type of behavior. Nevertheless, this variability allows us to associate the modulation of activity in the SPA with the computation of social prediction in any social situation and rule out the possibility that it is specific to a type of behavior. Future studies could build on our approach by distinguishing the predictability of social agents interacting with other social agents or with nonsocial items.

Together, our approach, built on theoretical debates about cross-species differences in TOM (*2*, *5*, *31*), provides new evidence for the existence in the last common ancestor to humans and macaques of a precursor neural architecture supporting computations associated with TOM in human TPJ (*31*).

## MATERIALS AND METHODS

### Data acquisition

#### Animals

Fourteen healthy rhesus macaque monkeys (*Macaca mulatta*; 13 males and 1 female) performed a set of free-watching tasks over a period of 6 months. All procedures were conducted under licenses from the UK Home Office in accordance with the UK Animals (Scientific Procedures) Act 1986. See table S4 for a detailed account of the number of runs per condition and per monkey.

#### Stimuli

Pictures and videos recorded at the breeding center and at the Oxford research colony were the basis of the video clips used in four experimental conditions. In addition, two other experimental conditions based on nonsocial stimuli were also used. Together, these six conditions and an awake resting-state acquisition (not included in this study) were presented in pseudo-randomized order. Four conditions described below have been used for the purpose of the current study. No more than three repetitions of a given condition were presented per day, the same condition was never repeated consecutively, and two different orders of presentation of the videos/pictures for a given condition were used to further limit habituation. For all conditions, the animals were not asked to fixate their gaze to conserve the most natural behavior. No reward delivery occurred during the presentation of stimuli. Reward was instead delivered in between two runs to maintain animal attention to the stimuli. The videos were selected to represent real-life events of the monkeys’ daily life. This means that the videos were not controlled for matching on some specific parameters (e.g., different types of social interaction). By introducing variety across videos for every single condition, we also reinforce the salience for the common attribute that defines each condition (e.g., the predictability of the social scenes).

First, we selected videos containing expected (e.g., grooming or playing; movie S1) and unexpected (e.g., unexpected deviation from grooming or playing; movie S2) social behaviors that were highly ecologically valid for the monkeys. The videos were presented for 5.5 s each and were combined in a 12-s block with 0.5 s of black screen before each video. Each block was followed by 10 s of rest (black screen). We presented three blocks of social unpredicted, three blocks of social predicted, and three blocks for each of their scrambled versions, respectively, in random order.

On the basis of a similar principle (deviation from expected situation), we created videos showing expected and unexpected object situation based on simple physical regularity (movies S3 and S4). In keeping with the social videos, object scenes showed events that could be unpredicted based on either location (object appearing at an unexpected location), identity (a new object appears), or movement (sudden change in movement patterns shown up to now). For instance, a video in which objects are falling at constant rate is considered predictable, while an unpredictable scenario would see this rate suddenly changed without an obvious cause. The timings for these conditions were the same than for the social prediction. We presented three blocks of object unpredicted situations, three blocks of object predicted situations, and three blocks for each of their scrambled version, respectively. This task was only done on 7 of the 14 monkeys.

For the social animation, we used stimuli from a previous study (*40*) showing abstract geometric shape behaving either socially or randomly. The timings for the social animation task are the same as that for the social prediction. There were six blocks of social interaction and six blocks of nonsocial interaction, with each block followed by 10 s of rest (black screen).

For the face localizer, the task followed a block design with each block of 12 s consisting of the presentation of eight images for 1 s each followed by 500 ms of black screen. A resting period of 10 s (black screen) was inserted between the face blocks. Each run was composed of three blocks of neutral faces, three blocks of emotional faces (aggressive or lip smacking), and six blocks of scrambled faces. This type of face localizer is known to capture face-responsive areas (*42*).

#### Awake and anaesthetized fMRI

The fMRI data were acquired in a horizontal 3-T MRI scanner with a full-size bore using a four-channel, phased-array, receive-only radio-frequency coil in conjunction with a local transmission coil (Windmiller Kolster Inc., Fresno, USA). The animals were head-fixed in a sphinx position in an MRI-compatible chair (Rogue Research, CA). fMRI data were acquired using a gradient-echo T2* echo planar imaging (EPI) sequence with the following parameters: resolution of 1.5 mm by 1.5 mm by 1.5 mm, 36 axial interleaved slices with no gap, repetition time (TR) of 2280 ms, echo time (TE) of 30 ms, and 130 volumes per run. Proton density–weighted images using a gradient-refocused echo sequence (TR = 10 ms, TE = 2.52 ms) were acquired as reference for offline image reconstruction.

Resting-state fMRI data and anatomical scans were collected under anesthesia for the same animals according to a previously used protocol (*17*). fMRI resting-state connectivity patterns are well conserved under anesthesia (*50*) and have been used for conducting human-macaque comparisons (*17*, *18*, *50*). Anesthesia was induced using intramuscular injection of ketamine (10 mg/kg) combined with either xylazine (0.125 to 0.25 mg/kg) or midazolam (0.1 mg/kg) and buprenorphine (0.01 mg/kg). Macaques also received injections of atropine (0.05 mg/kg), meloxicam (0.2 mg/kg), and ranitidine (0.05 mg/kg). Anesthesia was maintained with isoflurane. Isoflurane was selected because it has been demonstrated that resting-state networks are still present using this agent for anesthesia (*50*). The anesthetized animals were placed in an MRI-compatible stereotactic frame (Crist Instrument) in a sphinx position within a horizontal 3-T MRI scanner with a full-size bore. The same coils as for awake scans were used for data acquisition. Whole-brain BOLD fMRI data were collected using the following parameters: resolution of 1.5 mm by 1.5 mm by 1.5 mm, TR of 2280 ms, TE of 30 ms, 36 axial interleaved slices with no gap, and 1600 volumes. Structural scans were acquired in the same session using a T1-weighted MP-rage sequence (no slice gap, resolution of 0.5 mm by 0.5 mm by 0.5 mm, TR of 2500 ms, TE of 4.01 ms, and 128 slices).

### Preprocessing

All data were preprocessed and analyzed using tools from the FMRIB Software Library (version 5.0.10) (*51*), the Advanced Normalization Tools (version 2.1.0), and the Connectome Workbench software (www.humanconnectome.org). We also used MATLAB (version R2016a; MathWorks Inc., Natick, MA, USA) and bash codes from the Magnetic Resonance Comparative Anatomy toolbox (MrCat; www.neuroecologylab.org) and custom-made codes.

#### Task-fMRI preprocessing

Task-fMRI data were preprocessed following a dedicated nonhuman primate fMRI processing pipeline as part of the MrCat toolbox. In short, after offline SENSE reconstruction of the EPI image (Windmiller Kolster Scientific, USA), motion-induced time-varying slice distortions were corrected using restricted nonlinear registration, first to a run-specific high-fidelity EPI, then to each animal’s T1w structural image, and finally to group-specific template in CARET macaque F99 space (*52*). Brain extraction, bias correction, and template registration of the T1w structural image were achieved in an interdependent iterative approach. The resultant high-fidelity removal of nonbrain tissue could be back-projected to the EPI following nonlinear registration. A nuisance regressor design matrix was created to account for volumes with excessive movement, signal variability associated with motion-induced distortion artifacts, and nonbrain noise components. For the video tasks, we did not use the regressors for the nonbrain component, as they were correlated with the timing of the task. Further steps were implemented using the FEAT toolbox. We performed spatial smoothing using a Gaussian of 3-mm FWHM (full width at half minimum) kernel, grand mean intensity normalization, and high-pass temporal filtering (Gaussian-weighted least-squares straight-line fitting, with sigma = 100 s).

To assess for a proper coverage of the brain by the coils, we calculated the temporal signal-to-noise ratio (tSNR) associated with our data. For each session of the social prediction study, we obtained the mean intensity of the preprocessed time series, divided by the SD of the intensities at each voxel, and averaged these images across sessions (fig. S8). The result demonstrated a good coverage of the brain and particularly the frontal cortex, which is usually known for its poor tSNR.

#### Resting-state fMRI preprocessing

The detailed preprocessing pipeline for the resting-state fMRI has been described elsewhere (*39*, *53*). Briefly, after reorientation to the same convention for all functional EPI datasets, the first volumes were discarded to ensure a steady radio frequency excitation state. EPI time series were motion-corrected using MCFLIRT (*54*). Brain extraction, bias correction, and registration were achieved for the functional EPI datasets in an interdependent iterative manner. The mean of each functional dataset was registered to its corresponding T1w image using rigid-body boundary-based registration [FLIRT (*54*, *55*)]. EPI signal noise was reduced in both the frequency and temporal domain. The functional time series were high pass–filtered with a frequency cutoff at 2000 s. Temporally cyclical noise, for example, originating from the respiration apparatus, was removed using band-stop filters set dynamically to noise peaks in the frequency domain of the first three principal components of the time series. To account for remaining global signal confounds, we considered the signal time series in white matter and meningeal compartments, and their confound parameters were regressed out of the BOLD signal for each voxel. Following this confound cleaning step, the time series were low pass–filtered with a cutoff at 10 s. The data were transformed to the surface space using the F99 template and spatially smoothed using a 2.8-mm FWHM Gaussian kernel while considering the folding of the cortex. Last, the data time series were demeaned to prepare for functional connectivity analyses.

### Analysis

#### Contrasts

For the awake fMRI, the first-level analysis was carried out using FEAT for each run (*56*, *57*). Simple generalized linear model designs were defined. For the social prediction task, we used four explanatory variables (EVs), accounting for the social expected scene, social unexpected scene, and one for each of their scrambled versions. The main contrast of interest was between social unpredicted versus social predicted. We defined one more contrast as the scrambled unpredicted versus scrambled predicted to control for activity related to visual features (e.g., motion and luminance). We used a similar approach for the object prediction. For the social animation task, we used two EVs representing the social and random conditions. The main contrast was defined as social versus random. We also defined two more contrasts as social animation versus rest and random animation versus rest to further investigate both conditions. For the face task, four EVs were used to account respectively for the neutral face blocks, the emotional face blocks, the neutral scrambled blocks, and the emotional scrambled blocks. The main contrasts were defined as face images versus scrambled images and emotional faces versus neutral faces.

In each task, on top of the main contrasts, we defined a control contrast to detect neural activation when an image or video was present on the screen compared to rest period to confirm whether the monkeys were engaged during the task. As the task did not provide reward to the animals, they could disengage and fall asleep. We therefore excluded runs in which this control contrast elicited no or limited activation in the visual cortex. This method excluded five runs for the social prediction task and three runs for the object prediction task.

We applied a gamma hemodynamic response function convolution with a phase of 0 s, an SD of 1.5 s, and a delay of 3 s and the same temporal filtering as for the data. The movement regressors previously described were also used as additional confounds.

In the second-level analysis, after registration to standard space, we pooled together runs from the same monkeys. A fixed-effect analysis was performed at the subject level. Last, a third-level analysis was carried out to obtain the results at the group level using FLAME 1 as mixed-effects analysis with a cluster-forming *z*-threshold of 3.1 and corrected for family-wise error (FWE) at *P* < 0.05. The *z*-thresholds were chosen according to previous literature (*58*), which advises using the threshold of 3.1 with Flame 1 mixed-effect to avoid false positives. To test for a potential overlap of object prediction with social prediction, we used a more liberal threshold at *z* = 2.3. When no complete overlap is expected, as here, this approach increases the sensitivity of the test, allowing more stringent inferences.

#### Conjunction

We verified the specificity of the modulation by the social prediction videos by performing a series of conjunction analysis at the group level. All conjunctions are performed according to previous literature (*59*). We defined an STS mask comprising the gray matter of the STS excluding the very posterior parietal portion to restrict the conjunction and set the cluster-forming threshold at *z* = 3.1 and *P* < 0.05. For the conjunction between object prediction and social prediction, we used only the same seven animals available in both datasets. Because no significant conjunction was found between the object and social prediction at the *z* > 3.1 threshold, we lowered the threshold to 2.3, as above, to increase the sensitivity and account for the smaller number of animals in this condition.

#### Comparison of mean uncorrected z-statistic

To further confirm that this result was not due to a thresholding effect, we conducted additional analyses. We defined an ROI around the coordinates found in an anterior study (*18*) (most similar connectivity profile to human TPJ) with a 5-voxel radius. First, we computed the mean uncorrected *z*-statistic across voxels in this ROI for our three conditions (social prediction, visual control, and object control). The SD is defined as the square root of the variance of the *z*-statistic. We performed a Wilcoxon signed-rank test between conditions and corrected for multiple comparison using the Bonferroni method. Second, we performed the same third-level contrasts as before but restricting the statistics to the rostral midSTS ROI as defined before. Because the extent of this ROI is quite small, we performed both cluster- and voxel-thresholding corrections.

#### Hemispheric and regional specificity

We also investigated the hemispheric specificity of the social prediction modulation by analyzing the same contrast with an ROI either on the left or on the right hemisphere as performed in the literature (*60*). The ROI was defined as a coronal mask (five slices) encompassing the whole STS at the level of the small ROI mentioned earlier, around the coordinates found in the anterior study (*18*). This ROI was defined to overcome the issue of thresholding by reducing the number of voxels and to enlarge the search area so that we could capture clusters even if they were overlapping the borders of the small ROI (accounting for interindividual differences).

The MPFC has also been identified as part of the social brain in macaques (*21*). Therefore, we conducted another ROI analysis targeting the ACC to restrict the statistics to this previously identified region (*21*). No activity modulation of the ACC by the social prediction was revealed with this analysis.

#### Resting-data fMRI analysis

For the anaesthetized resting-data fMRI, in each monkey individually, we identified bilateral face patches from peak activation at the second-level analysis and based on the definitions of a previous study (*27*). We obtained the MF and ML in all monkeys; the AL, AF, and AM in 13 monkeys; and the PL in 12 monkeys. When the face patch was present on only one hemisphere, we defined the opposite hemisphere face patch as its symmetric voxels. We carried on the analysis on the 12 monkeys where we could find all the face patches in at least one hemisphere. Each face patch location was mapped to surface space, and an ROI was made of a circle of 2-mm geodesic distance, giving all ROIs the same size. We followed the same procedure for the SPA and defined an anterior SPA ROI that was part of the same cluster but could be found in all monkeys, insuring that we cover the entirety of the modulation location. We extracted the time series of each of these ROIs (six for face patches and two for social prediction) and computed their correlation with time series of the whole brain. We also performed a partial correlation where we regressed out the mean time series of all face patches from the SPA and the time series of the SPA from the face patches to obtain their specific connectivity. We then computed the correlation of these more specific time series to the whole brain. We therefore obtained two maps describing how each ROI connects to the rest of the brain for each monkey using both full correlation and partial correlation. We merged all monkeys for each seed and performed a nonparametric permutation inference using PALM (*61*) and performing the maximum number of permutations (in this case, sign flipping for a one-sample *t* test). Clusters were defined with the threshold-free cluster enhancement (TFCE) method, which enhances the cluster-like structures but keeps the voxel dimension of the data, and were corrected for multiple comparison using the FWE method.

For visualization, some of the results were projected onto the F99 surface using tools from the HCP Workbench and the inflated surfaces from a published study (*62*) (fig. S7).

### Human data

For the face task, we used the Neurosynth platform [created and maintained by T. Yarkoni, supported by National Institutes of Health (NIH) award R01MH096906] for automated meta-analysis that we probed with the word “faces.” The resulting meta-analysis map from 864 studies was then *z*-stats thresholded at 2.3 and projected onto a standard MNI (Montreal Neurological Institute) surface. The map is corrected using a false discovery rate (FDR) approach, with an expected FDR of 0.01.

For the resting-state human study, data were provided by the HCP, WU-Minn Consortium (principal investigators: D. Van Essen and K. Ugurbil; 1U54MH091657) funded by the 16 NIH Institutes and Centers that support the NIH Blueprint for Neuroscience Research and by the McDonnell Center for Systems Neuroscience at Washington University. We specifically used the group-average structural and functional MRI data from the HCP S1200 data release (March 2017). This dataset, available online at www.humanconnectome.org, allowed us to access task-related data but also resting-state connectivity network and atlases. The connectivity of TPJ was obtained from an ROI of 2-mm geodesic distance around the TPJ coordinates defined as in a previous study (*63*).

### Replication and TUS

One year after the first acquisition batch, we were able to acquire additional data for four animals (T2, T3, T4, and an additional monkey V1). Therefore, we conducted a replication study using 6 sessions for each of the conditions per animal (social prediction, 24 sessions; visual control, 24 sessions; and object control, 24 sessions). An additional animal was excluded because of the high level of head movement. We followed the exact same procedure, except for some technical acquisition and analysis details that we describe here. Data were collected with a 3-T MRI scanner with a full-size bore, and we used the four-channel, phased-array, receive-only radio-frequency coil in conjunction with a local transmission coil (Windmiller Kolster Inc., Fresno, USA). We used the exact same acquisition protocol. Concerning the analysis, we restrained our analysis to two levels because of the limited amount of data and because this is the most commonly used approach when having the same number of sessions for each animal. At threshold level 3.1, we did not obtain any significant result, but this was expected considering the lower amount of data. Therefore, we lowered the threshold to 2.3 and performed the same conjunction analysis and calculated the mean uncorrected *z*-statistic across voxels in this ROI as in the initial study.

We performed TUS on the same macaques used for the replication just before the fMRI free-viewing task to assess whether the FEF, through its attentional or oculomotor activity, could explain the modulation by social prediction in the SPA. The FEF is involved in attention and oculomotor movement such as saccades (*64*, *65*); its activity at rest is correlated with STS activity (*17*, *38*) and was also revealed in our social prediction analysis. As a control region, we stimulated the ACC, which is involved in the extended social brain. The impact of TUS on FEF and ACC and their consequence on behavior have already been demonstrated (*53*, *64*–*66*). We also collected control data for which no stimulation was performed (note that these are the data used in the replication). For these three stimulation conditions, we acquired six runs per monkey per condition (social prediction, visual control, and object control). Control days were interleaved with TUS sonication days. TUS was performed using the same protocol as previously published (*66*, *67*), adapting the focal depth of the transducer to the desired coordinates. A sequential stimulation was performed to target the left and right FEF (*67*). A unique stimulation was performed on the midline for achieving a bilateral ACC stimulation (*66*).

Briefly, a single-element ultrasound transducer was used for 40 s. It was positioned with the help of the Brainsight neuronavigation system (Rogue Research) so that the focal spot was centered on the targeted brain region, namely, the FEF on the anterior bank of the arcuate sulcus (left FEF MNI coordinates ± SD: *x* = −14.4 ± 0.9, *y* = 4.9 ± 2.5, *z* = 13.3 ± 1.4; right FEF: *x* = 15 ± 1.2, *y* = 4.2 ± 1.6, *z* = 11.8 ± 1.5) and the controlled region: the ACC rostral to the genu of the corpus callosum (MNI coordinates ± SD: *x* = 0 ± 0.9, *y* = 15.5 ± 1.5, *z* = 6.5 ± 1.0). The ultrasound wave frequency was set to the 250-kHz resonance frequency, and 30-ms bursts of ultrasound were generated every 100 ms (duty cycle, 30%) with a digital function generator (Handyscope HS5, TiePie engineering, Sneek, The Netherlands). Overall, the stimulation lasted for 40 s. A 75-W amplifier (75A250A, Amplifier Research, Souderton, PA) was used to deliver the required power to the transducer. A TiePie probe (Handyscope HS5, TiePie engineering, Sneek, The Netherlands) connected to an oscilloscope was used to monitor the voltage delivered. Note that one FEF session for one animal was conducted with a higher intensity and longer duration (60% duty cycle instead of 30% for 60 s instead of 40 s), which resulted in a localized skin trauma. The recorded peak-to-peak voltage was constant throughout the stimulation session. Voltage values per session ranged from 128 to 136 V and corresponded to a peak negative pressure ranging from 1.15 to 1.29 MPa, respectively, as measured in water with an in-house heterodyne interferometer (*68*). On the basis of a mean 66% transmission through the skull (*69*), the estimated peak negative pressures applied were between 0.75 and 0.85 MPa at the target in the brain.

fMRI data acquisition, preprocessing, and analysis were performed as described for the replication. To compare control condition contrasts with stimulation condition contrasts, we performed a two-sample paired *t* test, regressing out the mean of each subject so that it would not interfere with the estimation of the difference between stimulation conditions. To assess that the stimulations had any effect, we compared a simple visual contrast (videos versus black screen) and a social contrast (social videos versus scrambled). Having established that stimulations did change some of the brain task-related modulation, we compared the contrast of interest: the social prediction. We used a whole-brain analysis and an ROI analysis. This ROI combined the left and right ROI defined for the hemispheric analysis, resulting in a coronal mask encompassing the whole STS bilaterally at the level of the small ROI mentioned earlier. This ROI was defined to overcome the issue of thresholding and interindividual difference.

Last, because the replication study involved three monkeys used in the initial study (T2, T3, and T4), we were able to evaluate the variability between studies. We compared our three main contrasts of interest: the social prediction, visual control, and object control using six sessions per animal for each condition, a two-level analysis, and a cluster threshold at 2.3. We performed a two-sample paired *t* test, regressing out the mean of each subject comparing the initial and replication study. No significant difference was observed in the midSTS. The only significant differences were the following: For the replication study, higher activation could be observed in the visual cortex for the social prediction contrast and in the frontal cortex for the visual contrast (fig. S9). No difference was observed for the object contrast.

## Notes

View/request a protocol for this paper from *Bio-protocol*.
