# Supplementary material for: Social prediction modulates activity of macaque superior temporal cortex
Source: Sci Adv. 2021 Sep 15;7(38):eabh2392. doi: 10.1126/sciadv.abh2392 (PMC8443173; doi:10.1126/sciadv.abh2392)
Supplement: Supplementary file 1 — Figs. S1 to S9 Tables S1 to S4 Legends for movies S1 to S4 [file sciadv.abh2392_sm.pdf]

Science Advances
AAAS

Supplementary Materials for

Social prediction modulates activity of macaque superior temporal cortex

Lea Roumazeilles*, Matthias Schurz, Mathilde Lojkiewiez, Lennart Verhagen, Urs Schüffelgen, Kevin Marche, Ali Mahmoodi, Andrew Emberton, Kelly Simpson, Olivier Joly, Mehdi Khamassi, Matthew F. S. Rushworth, Rogier B. Mars, Jérôme Sallet*

*Corresponding author. Email: learoumazeilles@gmail.com (L.R.); jerome.sallet@inserm.fr (J.S.)

Published 15 September 2021, *Sci. Adv.* **7**, eabh2392 (2021)
DOI: 10.1126/sciadv.abh2392

The PDF file includes:

Figs. S1 to S9
Tables S1 to S4
Legends for movies S1 to S4

Other Supplementary Material for this manuscript includes the following:

Movies S1 to S4

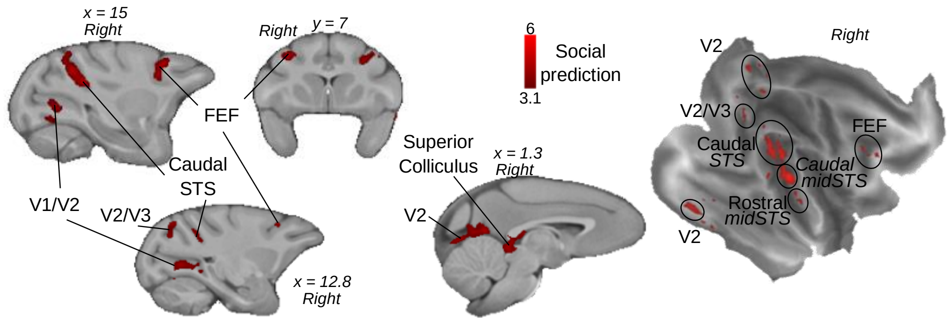

Fig. S1. Social prediction contrast. Group contrast of unexpected versus expected social situation also revealed activity in Frontal Eye Field (FEF), Superior Colliculus, Visual area V1/V2 and Visual area V2/V3 (extending into V4) as seen on F99 slices (left) and flat map (right) (n=14, cluster-corrected at z>3.1, p<0.05 FWE corrected).

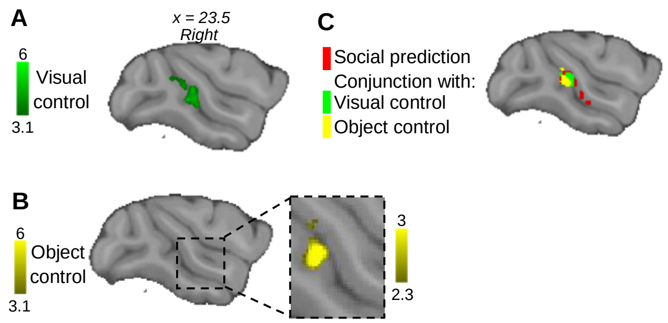

Fig. S2. Control conditions. (**A**) Visual control: group contrast of unexpected versus expected scrambled social scenes revealed activity in caudal midSTS only (n=14, cluster-corrected at z>3.1, p<0.05 FWE corrected). (**B**) Non-social prediction (object) control: group contrast of unexpected versus expected object scenes revealed no activity (n=7, cluster-corrected at z>3.1 and p<0.05 FWE corrected). At lower threshold (insert), the contrast revealed activity in caudal midSTS only (cluster-corrected at z>2.3, p<0.05 FWE corrected). (**C**) Conjunction results between the social prediction contrast and the control contrasts (cluster-corrected at z>3.1 for visual feature control and at z>2.3 for object control, p<0.05 FWE corrected).

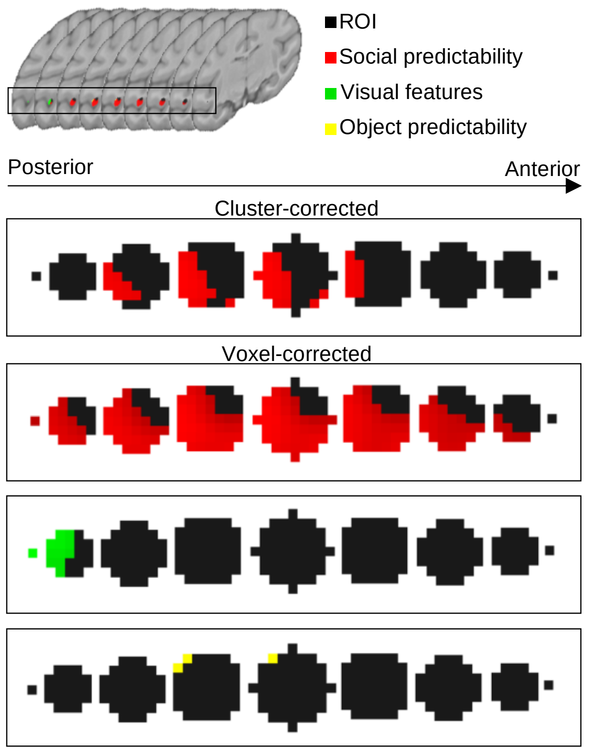

Fig. S3. ROI analysis cluster and voxel corrected. Representation of the midSTS ROI from previous study (18), from posterior to anterior coronal slices. When cluster-corrected (z>3.1) only the social prediction contrast was significant. When voxel-corrected (p<0.05), a few voxels in the two other controls were significant.

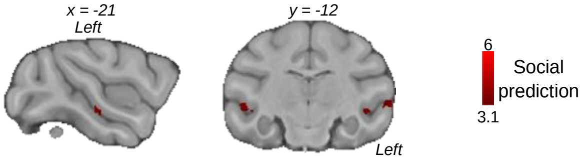

Fig. S4. Hemispheric analysis. Social prediction: group contrast of unexpected versus expected social situation restricted on a rostral midSTS ROI revealed activity in rostral on the left hemisphere midSTS (n=14, cluster-corrected at z>3.1, p<0.05 FWE corrected).

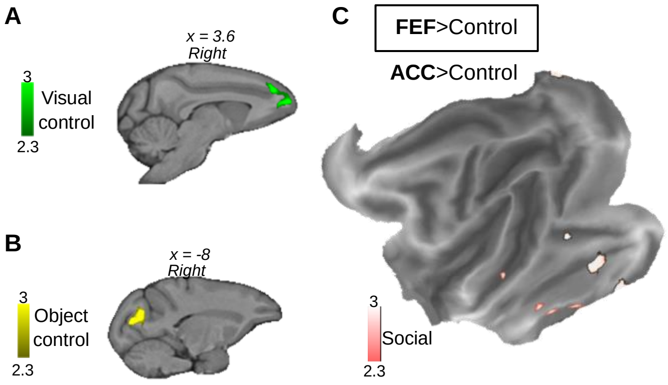

Fig. S5. Replication of the control conditions and TUS modulation. (**A**) Visual control: group contrast of unexpected versus expected scrambled social (n=4, cluster-corrected at z>2.3, p<0.05 FWE corrected). (**B**) Non-social prediction (object) control: group contrast of unexpected versus expected object scenes (n=4, cluster-corrected at z>2.3 and p<0.05 FWE corrected). (**C**) Two-sample paired t-test for higher activation in FEF stimulation condition (black-outline) or ACC (no outline) compared to control for the group contrast of social videos versus scrambled videos represented on a flat map (n=4, cluster-corrected at z>2.3, p<0.05 FWE corrected).

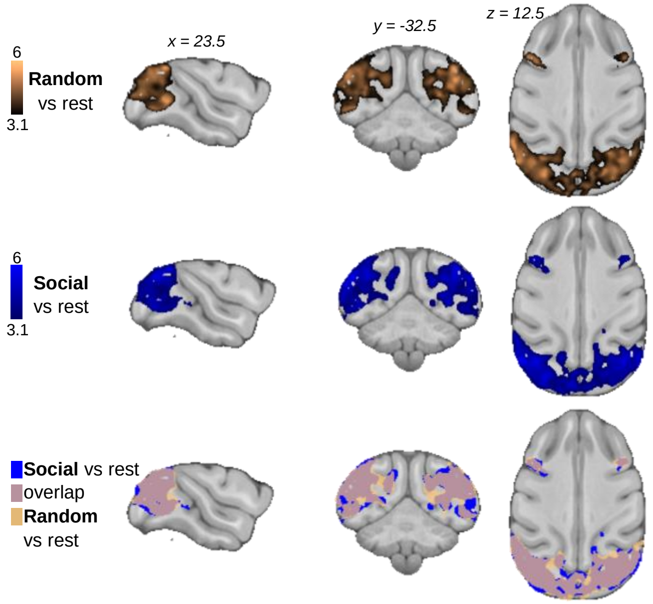

Fig. S6. Social animation. Group contrast of abstract shape videos versus rest blocks when acting either randomly (copper) or socially (blue) and their overlap (n=14, cluster-corrected at $z>3.1$, $p<0.05$ FWE corrected).

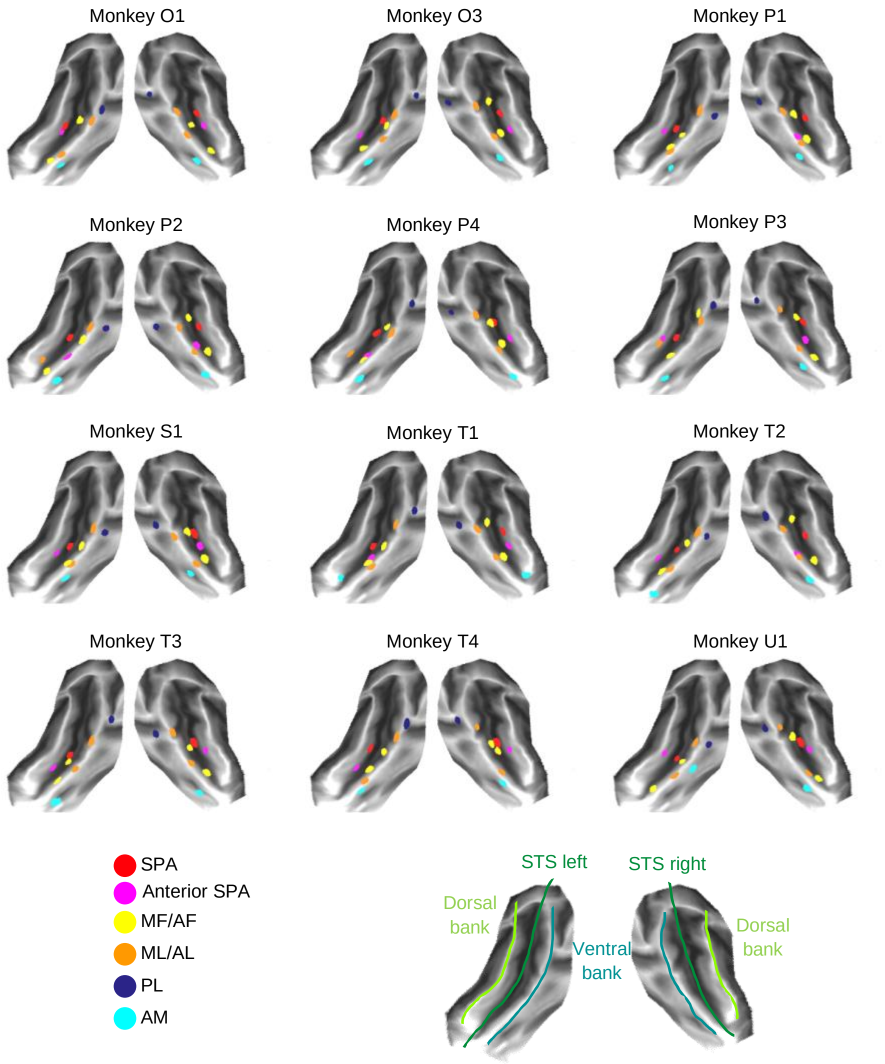

Fig. S7. Peak activities for individual macaques. Peak activity for the SPA and for the face patches represented on a flat F99 surface showing the STS with its dorsal and ventral bank.

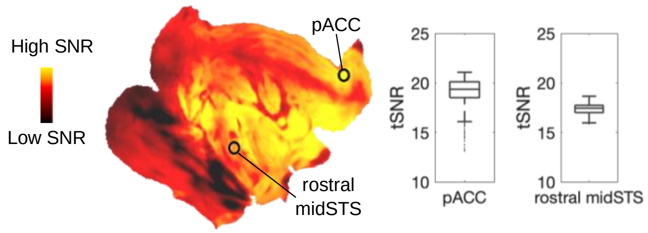

Fig. S8. Temporal signal to noise ratio (tSNR). Flat map representing the right hemisphere tSNR calculated across all sessions of the social prediction task and box plot of tSNR in two regions of interest: the perigenual anterior cingulate cortex (pACC) and the rostral middle superior temporal sulcus (midSTS). On each box, the central mark indicates the median, and the bottom and top edges of the box indicate the 25th and 75th percentiles, respectively. The whiskers extend to the most extreme data points not considered outliers. Outliers are represented with dots.

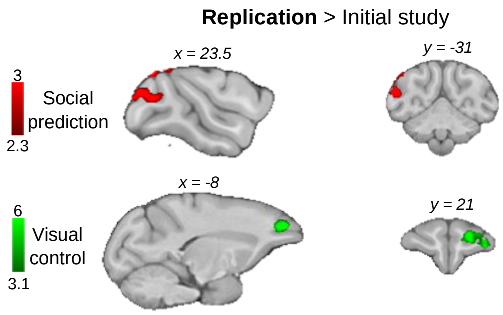

Fig. S9. Variability in the replication assessed in the three same monkeys. Two-sample paired t-test for higher activation in the replication compared to initial study for the social prediction contrast and visual control (n=3, cluster-corrected at $z>2.3$, $p<0.05$ FWE corrected).

	Social prediction			Visual control			Object control		
	**x**	**y**	**z**	**x**	**y**	**z**	**x**	**y**	**z**
midSTS rostral	24.6	-11.6	-2.51						
midSTS caudal	22.6	-16.6	3.02	22.1	-17.1	1.51	23.1	-17.1	1.01
FEF	15.1	7.04	16.6						
V2/V3	8.05	-37.2	18.1						
V1 lateral	13.6	-33.2	1.01						
V2 medial	1.51	-31.2	6.54						
Superior colliculus	2.52	-20.1	1.51						

Coordinates given for the right hemisphere in mm in F99 standard space (Social prediction and visual control: n=14, cluster-corrected at z>3.1 and p<0.05 FWE corrected, object control: n=7, cluster-corrected at z>2.3 and p<0.05 FWE corrected).

Table S1. Peak activation coordinates of social prediction and controls at the group level.

	Social prediction		
	x	y	z
midSTS rostral	21	-12.6	-4.9
midSTS caudal	20.1	-20.6	4.02
FEF	-18.1	7.04	20.1
Parietal area PG	10.6	-37.2	19..6
aSTS	-28.2	-10.1	-8.05

Coordinates given in mm in F99 standard space (n=4, cluster-corrected at z>2.3 and p<0.05 FWE corrected).

Table S2. Peak activation coordinates of social prediction at the group level for the replication study.

	Right			Left		
	**x**	**y**	**z**	**x**	**y**	**z**
AM	22.1	-1.5	-16.3	-20.5	-0.7	-14.7
AF	22.6	-2	-10.1	-20.5	-5.2	-9.8
AL	24.6	-0.9	-9.5	-22.5	-1.17	-10.8
MF	25.2	-15.6	-0.5	-22.0	-14.6	-4.6
ML	30.7	-16.1	4.5	-27.6	-17.2	2.5
FEF	21.1	10.6	8.6	-21.1	10.6	6.54

Coordinates given for the right and left hemisphere in mm in F99 standard space (n=14, cluster-corrected at z>3.1 and p<0.05 FWE corrected).

Table S3. Peak activation coordinates of the face localizer at the group level.

Monkey ID	Age (years)	Weight (kg)	Social prediction	Object prediction	Social animation	Faces	*Replication* Social Prediction	*Replication* Object Prediction
O1	13	12	10	-	11	13	-	-
O2	13	12	4	-	7	9	-	-
O3	13	12	9	-	6	13	-	-
P1	12	11	11	-	11	12	-	-
P2	12	11.5	8	-	9	9	-	-
P3	12	11.5	10	-	10	9	-	-
P4	12	11.5	10	-	13	11	-	-
S1*	9	7.5	8	8	11	10	-	-
T1	8	11.5	5	10	7	10	-	-
T2	8	14	10	10	11	9	6	6
T3	8	12	10	9	11	9	6	6
T4	8	13	8	11	9	10	6	6
U1	7	13	11	10	9	10	-	-
U2	7	11	10	8	12	11	-	-
V1	-	-	-	-	-	-	6	6
Total			124 (n=14)	66 (n=7)	137 (n=14)	145 (n=14)	24 (n =4)	24 (n=4)

*Female

Table S4. Detail of the monkeys and number of runs per subjects and per conditions selected for analysis.

Movie S1. Videos used in the predicted social situation condition. Each video is 5.5 seconds, six videos are combined in one movie.

Movie S2. Videos used in the unpredicted social situation condition. Each video is 5.5 seconds, six videos are combined in one movie.

Movie S3. Videos used in the object control showing the predicted object situation condition. Each video is 5.5 seconds, the six videos are combined in one movie.

Movie S4. Videos used in the object control showing the unpredicted object situation condition. Each video is 5.5 seconds, the six videos are combined in one movie.
